# Application of Computational Method in Designing a Unit Cell of Bone Tissue Engineering Scaffold: A Review

**DOI:** 10.3390/polym13101584

**Published:** 2021-05-14

**Authors:** Nur Syahirah Mustafa, Nor Hasrul Akhmal, Sudin Izman, Mat Hussin Ab Talib, Ashrul Ishak Mohamad Shaiful, Mohd Nazri Bin Omar, Nor Zaiazmin Yahaya, Suhaimi Illias

**Affiliations:** 1 Faculty of Engineering, School of Mechanical Engineering, Universiti Teknologi Malaysia, Johor Bahru, Johor 81310, Malaysia; nsyahirah83@graduate.utm.my (N.S.M.); izman@utm.my (S.I.); mathussin@utm.my (M.H.A.T.); 2 Faculty of Mechanical Engineering Technology, Universiti Malaysia Perlis, Arau 02600, Malaysia; mshaiful@unimap.edu.my (A.I.M.S.); nazriomar@unimap.edu.my (M.N.B.O.); zaiazmin@unimap.edu.my (N.Z.Y.); suhaimi@unimap.edu.my (S.I.)

**Keywords:** numerical analysis, computational method, tissue engineering scaffold design, mechanical strength, simulation software

## Abstract

The design of a scaffold of bone tissue engineering plays an important role in ensuring cell viability and cell growth. Therefore, it is a necessity to produce an ideal scaffold by predicting and simulating the properties of the scaffold. Hence, the computational method should be adopted since it has a huge potential to be used in the implementation of the scaffold of bone tissue engineering. To explore the field of computational method in the area of bone tissue engineering, this paper provides an overview of the usage of a computational method in designing a unit cell of bone tissue engineering scaffold. In order to design a unit cell of the scaffold, we discussed two categories of unit cells that can be used to design a feasible scaffold, which are non-parametric and parametric designs. These designs were later described and being categorised into multiple types according to their characteristics, such as circular structures and Triply Periodic Minimal Surface (TPMS) structures. The advantages and disadvantages of these designs were discussed. Moreover, this paper also represents some software that was used in simulating and designing the bone tissue scaffold. The challenges and future work recommendations had also been included in this paper.

## 1. Introduction

An engineered tissue can be a huge aid in the future, especially in clinical application. The relationship that brings life sciences and engineering together as an application to be a great help in understanding the structure and function of a mammalian tissue can be described as tissue engineering [1]. Not only offering help in understanding the structure and function of tissue of a human being, but it is also helping the researchers to understand the necessity of developing an engineered tissue. An engineered tissue can help in restoring, maintaining, repairing and improving the damaged tissue’s condition, which is caused by numerous diseases such as disabilities and injuries [2]. Zhang et al. stated in their paper that the current implementation of tissue engineering had faced many issues, including ethical and technical issues [3]. Despite many challenges faced in the field of tissue engineering, it is a fast-paced developing field since it can be a great help in providing treatments that can generate most of the tissue and organ of the human being [3].

One of the components that is crucially needed to be studied is the scaffold of the engineered tissue. This is due to the function of the scaffold that provides a suitable environment and structure in order to enable the cells to attach, proliferate, differentiate and secrete their own extra-cellular matrix (ECM) [3]. It is important to ensure the scaffold to have a proper environment and structure so that it promotes a good rate of the formation of tissue. In order to produce an appropriate scaffold, it must be ensured to possess a few characteristics so that it will not be harmful to the body. The characteristics included bio-compatibility, biodegradable, bioactivity, scaffold architecture and mechanical properties. Turnbull et al. reported that the manufacturing of a scaffold should be compatible with the human body so that it will not trigger any immune response while it is implanted in the body [4]. In order to comply with these conditions, the materials used in manufacturing the scaffold should non-toxic and easy to eliminate from the body.

Another important feature in manufacturing the scaffold is that the degradation rate of the scaffold is needed to be properly controlled. This is to ensure that the scaffold does not suffer mechanical failure. In addition, the scaffold’s structure and architecture need to be considered when manufacturing a scaffold because it provides viability and encourages tissue ingrowth. The human body is a very sensitive creation where all the parts are subjected to a certain value of strength and provide sufficient endurance of pressure. Therefore, it is crucial that the mechanical properties of the manufactured scaffold achieve the same or properly adjusted to the original tissue so that it can have no negative effect on the host tissue [5,6]. All these features will help in promoting tissue growth and avoiding a negative response of the immune system if the scaffold can be produced in a proper manner [4].

The ‘trial-and-error’ method has been adopted by most common researchers in enhancing the tissue engineering field. The method which involved the modification of the current or existing design of a scaffold can cause many unwanted factors. This conventional method is very expensive and does not have a precise control due to the repeatable modification. It is also time-consuming since the production of an improved model of the scaffold will take too long. Therefore, a computational approach needs to be used.

Besides that, although much research has been done on a scaffold of tissue engineering computationally, there is still a lack of research that focuses on fluid properties and designs of the scaffolds. In addition, the designs and fluid properties of a scaffold play a crucial role in facilitating the growth of the bone tissue.

The porosity and mechanical strength of the scaffold have an inversely proportional relationship. However, bone scaffold needs to be manufactured porously in order to enable cell proliferation and transportation of nutrients, oxygen and metabolites in the blood [7,8,9]. Yet, up to this date, there is very little research done that can computationally produce a scaffold with good design and possessing excellent mechanical and fluid properties, simultaneously.

Therefore, various great efforts have been done by many researchers in order to help in producing a scaffold model that possesses all of the ideal characteristics. One of the ways to develop a scaffold model that can cater to the needs to encourage a high rate of tissue formation is by using computational methods, which consist of simulating, modelling and 3D printing techniques. Current research studies show that the computational method has been a great help in order to expedite the implementation of tissue engineering in the near future.

### 1.1. Bone Tissue Engineering

The most major structural and connective tissue of the body is bone tissue [10]. There are two types of bones that can be identified, which are cortical bones and cancellous bone. The outer part of the cone that is denser and has low porosity is called cortical bone, while the inner part and spongy-like material is called cancellous bone. The porosities of the cancellous bone should be in the range of 50% to 80% [10,11,12,13]. The bone is one of the parts that is having high mechanical strength. A cortical bone possesses a high modulus of elasticity and compressive strength as compared to the cancellous bones [14]. Although bone has high mechanical strength, bone can be subjected to many traumas and diseases such as injuries. Therefore, researchers have come to a solution which to produce regenerative medicine in terms of tissue engineering. Despite many organs that can be regenerated by the method of tissue engineering, bone tissue engineering is a widely studied field.

A large bone defect is treated with the current conventional method, which is by using autografting. Autografting technique required the usage of bone from a non-load-bearing site of the patient to be transplanted into the damaged part [10]. Nowadays, various researchers have led to the implementation of bone tissue engineering in the future since it can overcome the problems faced by current clinical treatments [15].

### 1.2. Scaffold of Bone Tissue Engineering

A biomaterial porous structure that helps in providing support and a suitable extra-cellular matrix is called a scaffold [16,17]. In designing a scaffold, one must consider the strength and porosity of the materials so that it will regenerate the properties of the bone that is comparable to the original bone. Nowadays, there are many applications of technology to develop a scaffold, such as additive manufacturing, which includes 3D printing. Additive manufacturing has provided a platform which helps in customizing and developing a suitable design that can be used in biomedical application.

The scaffolds must be designed and developed based on a few characteristics that will provide the best condition for the bone to regenerate. The characteristics include biocompatibility, bioactivity, biodegradability, mechanical properties and scaffold architecture [18]. Biocompatibility can be defined as non-toxic and non-inflammatory so that it will not bring harm to the body [5,6,19]. A biodegradable scaffold should be able to eliminate itself from the body easily once the tissue has fully restored. Adequate mechanical properties should be possessed by the scaffold so that it will be able to withstand any forces and loads during the restoration time in the implantation site [19].

Designing a scaffold with a proper architecture is important due to it will affect the mechanical and biocompatibility properties of the scaffold [20,21]. Scaffold architecture should be able to provide a large surface area to volume ratio so that cell migration can occur. The porosity must be sufficient so that it will allow cell and nutrition migration for the restoration of the tissue. However, it must not compromise with the mechanical strength of the scaffold [22].

### 1.3. Significance of Computational Method in Bone Tissue Engineering

The successful production of bone tissue engineering scaffold can help to contribute to ensuring the tissue formation goes smoothly, especially bone tissue. This is due to the computational method to help in generating precise properties of the scaffold. Besides that, the usage of the computational method, which is the simulation aimed to provide good support in implementing the usage of tissue engineering in the future, especially in clinical application. This is because the simulation can help in reducing the intervention of humans in manufacturing the appropriate scaffold model. The risks will be minimized due to the involvement of automation that will eventually produce fewer damaged organs.

## 2. Computational Method in Designing a Scaffold

Computational modelling has been the most common approach that had been done by the researchers. This is due to its ability to simulate the behaviour of the scaffold under certain loadings. In addition, it is proved to reduce time and experiments since it is not time-consuming and cheaper [23].

Moreover, the computational modelling technique has been adopted by the researchers in order to improve the performance of scaffolds while maintaining certain important parameters [3]. It is also a great predictive tool that can help in predicting the scaffold properties before manufacturing them. Some uncommon properties, such as stress-strain distribution, can also be predicted by using the computational method.

According to Bocaccio et al., the computational method has allowed the approximation of how the mechanical environment is affecting the differentiation of tissue and bone regeneration [24]. It also helps in understanding the mechanisms that will enhance the reliability of the function of scaffolds. Zhang et al. stated that the function of computational modelling includes designing and simulation that had been a great aid in 3D printing technique [4].

Recently, the usage of the computational method in assessing the properties of a scaffold’s structure has been progressively studied. With the aid of computational programs, such as Finite Elements Analysis (FEA), the properties of the scaffold can be easily predicted. Thus, it helps in reducing the time and energy to find the most feasible scaffold by eliminating the modification step of an existing scaffold.

### 2.1. Unit Cell of a Scaffold Structure

The unit cell is the basic structure of a scaffold. It can be divided into two types of designs which are non-parametric and parametric. Non-parametric designs consist of a unit cell which is designed by using structural and geometric shape. Meanwhile, the parametric designs have to be produced by using specific algorithms. There are many advantages and disadvantages regarding each design which will be furthered discussed.

### 2.2. Non-Parametric Design

A non-parametric design is a simple structure that is designed based on geometry. The most common non-parametric designs are the circular, cubic and honeycomb designs as such in Table 1. However, there are a lot of other designs, which are produced based on certain geometries, such as hexagonal and octet. There are many advantages of these designs as compared to the parametric designs. One of them is that the non-parametric designs are easy to be produced since it does not engage to any specific algorithms. In addition, there are many ways that can be used to fabricate the designs. These designs are mostly being fabricated via subtractive manufacturing such as machining. However, since most of the researchers are looking forward to utilizing additive manufacturing, the production of these designs is very much possible to pursue, especially by using Selective Laser Melting (SLM) additive manufacturing. The characteristics of non-parametric designs can be described, as in Table 1.

#### 2.2.1. Circular Design

Many studies have been done that adopted circular pore shape as their scaffold model. This is due to the ability of the circular pore to avoid stress concentration point; therefore, it relatively would possess a high bearing stress capacity [18]. The study conducted by Sun et al. showed that the circular shape produced a more uniform axial deformation. Thus, it gives a smaller strain risk when subjected to a uniform stress concentration [25]. Bocaccio et al. suggested that the circular design exhibits a greater Young’s Modulus when they are subjected to a certain amount of pressure [26].

In terms of the porosity of the scaffold, the pore size of the scaffold plays an important role in determining the mechanical properties of the scaffold. Boccaccio et al. suggested that the circular pore demonstrated a certain amount of mechanical properties when its porosity distribution law is varied [27]. A circular pore with a low amount of porosity also helps in ensuring a high amount of mechanical strength [28]. Although the porosity amount of a circular-shaped pore is high, its mechanical strength was lower in a study carried out by Jahir-Hussain et al. [29]. Therefore, the circular-shaped pore needs to possess a high porosity amount so that it can enhance mechanical and morphological properties [30]. Gomez et al., in their study, described that the circular-shaped scaffold needs to possess a porosity amount in the range of 70–90% in order to obtain a high mechanical strength [31].

#### 2.2.2. Square Design

Although the square-shaped designs have a high-stress concentration region, they are also still relevant to be studied by the researchers since they possess a high mechanical strength while maintaining an adequate amount of porosity. In order to avoid this problem, researchers came out with a solution where they modified the square scaffold by adding a few struts. The struts help to improve the stiffness of the porous structure as well as reduces the stress concentration at the joints [37]. In a study conducted by Jahir-Hussain et al., they varied the pore shape of the scaffold, resulting in high mechanical strength but a low amount of porosity [29]. When the porosity of the scaffold is increased to be more than 50%, the square-shaped scaffold can exhibit mechanical properties similar to that of the host tissue [36]. Habib et al. modified the square-shaped scaffold by increasing the porosity but could maintain the mechanical properties of the scaffold [19]. The failure mechanism of a square-shaped scaffold is that it failed in the unidirectional failure according to the direction of loading subjected to it.

#### 2.2.3. Honeycomb Design

The honeycomb structure was designed based on the hexagonal prismatic wax cells, which are built by honey bees. In the engineering field, the honeycomb structure was first introduced to the aerospace discipline. However, it gets the attention of the other fields’ researchers, including the biomedical field, since it can be found naturally in biomedical structure. Moreover, it is light-weighted with a high amount of stiffness and porosity.

By using Finite Element Analysis, the mechanical properties of the honeycomb structures can be simulated. The Young’s Modulus of the honeycomb structure can be controlled by varying the porosity of the structures. This would cause the honeycomb design to be able to fit in between cortical and cancellous bone properties [21,45]. However, the honeycomb tends to fail in multi-directions when it is exposed to certain loadings [44]. There are a few designs that were generated by modifying the original shape of the honeycomb structure. The new design of the honeycomb structure has the ability to demonstrate the mechanical properties of a cancellous bone [46].

In conclusion, we can say that a non-parametric design can still be adopted into various research works due to its ability to demonstrate the desired properties of a bone tissue engineering scaffold. However, there is a need to modify the design in order to match the properties of the host tissues. A circular design exhibits an excellent characteristic in terms of fewer stress concentration points as compared to other non-parametric designs. Meanwhile, a square-shaped pore promotes a high rate of cell proliferation due to its ability to possess a high amount of porosity. The honeycomb structure has the ability to maintain excellent mechanical stability by reducing the risk of scaffold shrinkage during cell growth.

### 2.3. Parametric Design

Since tissue engineering is highly related to the usage of additive manufacturing (AM), the researchers tend to shift from using simple structure to using complex structure since the AM technology has the ability to produce a complex structure [47]. The simpler shape is more likely to face some issues such as strut thickness [48], interface mismatch [49] and surface smoothness [50]. According to Chen et al., there are two main methods that were used to generate a parametric structure, named Triply Periodic Minimal Surfaces (TPMS) and Voronoi Tessellation, as shown in Table 2 [8].

#### 2.3.1. Triply Periodic Minimal Surfaces (TPMS)

Triply Periodic Minimal Surfaces (TPMS) is a smooth infinite and non-self-intersecting periodic structure in three principal directions associated with crystallographic space group symmetry [68,69]. In 1865–1883, Schwarz and Neovius had introduced some TPMS structures, which are Schwarz P (Primitives), Schwarz D (Diamond), Schwarz H (Hexagonal) and Neovius. In 1970, Schoen described the most popular TPMS structure, which is the Gyroid and also a few other TPMS structures [70]. The most common TPMS structures that were studied by the researchers are Gyroid, Diamond and Primitives [71]. This is because the structures can be easily found in nature, such as butterfly wing scales and sea urchins [72]. TPMS structures are likely to be favoured by the researchers since it promotes higher cell attachment, migration and proliferation as compared to the scaffold with sharp edges [47,73].

TPMS structure can be classified into two types which are skeletal TPMS and sheet TPMS. Most of the researchers tend to assess the properties of TPMS via the sheet typed TPMS. Therefore, there is a lack of research on the skeletal TPMS structure. In research conducted by Barba et al., they found that the Gyroid skeletal TPMS shows a feasible design of a scaffold, which is superior in terms of manufacturability, mechanical properties and bone ingrowth [74]. However, Cai et al. stated that the compressive strength of the skeletal TPMS is much lower as compared to the sheet TPMS [75].

Meanwhile, the sheet TPMS structure is widely assessed in the literature as it shows a superior design as compared to the skeletal TPMS. There are many types of TPMS structures that can be generated through a mathematical equation that controls the TPMS structure. By using a few software such as Minisurf, the TPMS structure can easily be generated [76]. The built-in equations can be tabulated in Table 3.

However, the most assessed sheet-TPMS structure in the previous studies are Schwarz P (Primitives), I-WP, Schwarz D (Diamond) and Gyroid. This is due to their ability to match the properties of the host tissue of cancellous and cortical bone. According to Bobbert et al., these structures are able to avoid stress shielding by possessing high yield stress and low Young’s Modulus [50]. These TPMS can be categorized into two main categories that are based on their deformation mechanisms, which are stretching surface and bending surface [68]. Schwarz P (Primitives) and I-WP belong to the stretching surface, while Schwarz D (Diamond) and Gyroid are the bending surface TPMS.

By using the computational method, which is adopting the Finite Element Analysis (FEA) to assess the properties of the TPMS scaffold, Shi et al. found out that the TPMS scaffold possessed excellent scaffold properties that are matched with the bone tissue properties [80]. In terms of porosity, Castro et al. had reported that the gyroid TPMS can be used in clinical practices in the bone tissue engineering field. They had carried out both numerical and experimental methods to assess the mechanical properties of two gyroids with 50% and 70% porosity, respectively [81]. In a research carried out by Yang et al., they found out that the Young’s Modulus of Schwarz P, Schwarz D, I-WP and Gyroid were matched to the Young’s Modulus of the cancellous bone. However, this only happened when they were subjected to a high amount of porosity [82]. In addition, the compressive properties of Schwarz P and I-WP was higher than the cancellous bone [54]. This fact is supported by Montezarian et al., when they also reported that the compressive strength was higher in the Schwarz P and I-WP structures than the cancellous bone [48,61]. Meanwhile, at a low amount of porosity in the range of 5–10%, the Schwarz P and Schwarz D scaffolds possessed Young’s Modulus properties similar to that of the cortical bone [83].

Most of the researchers tend to compare the mechanical properties of these TPMS structures in order to determine the suitable application of the structure in clinical practice in the future. For example, Afshar et al. reported that the Schwarz P structure showed better mechanical properties as compared to the Schwarz D structure [84]. In addition, Maskery et al. also had stated the same conclusion since they found that the stretching surface TPMS has twice the Young’s modulus of bending surface TPMS by using Finite Element Analysis [85]. In order to find the mechanical properties, Finite Element Analysis showed the failure mechanism while simulating the behaviour of the structure. In their research, Maskery et al. had suggested that the stretching surface TPMS failed because the stress concentration region was located at the Schwarz P neck, which is situated at the top surface of the whole structure [85]. This has shown that the structure would start to fail layer by layer when it is subjected to loadings [86,87]. However, the bending surface TPMS would start to fail once the scaffold is subjected to ultimate pressure due to the uniform stress distribution in bending surface TPMS by showing a shear band [48,54,84,88,89]. From these studies, we can see that the stretching structure would fail due to the axial deformation while the bending structure would fail once the shearing linkages appear on the structure. Thus, the stretching structures possess a high capacity of load-bearing as compared to the bending structures when a uniaxial loading is subjected to them [68].

Although the porosity of the TPMS structure can be predetermined by varying certain parameters in the mathematical equation, the actual porosity amount of the structure was consistent with the TPMS design. This is due to some studies showed that the solid structure such as cube has lower mechanical properties as compared to the TPMS structure. In a study conducted by Zaharin et al., they discovered that the gyroid structure is mechanically better than the cubic structure [58]. The strut-based structure is also happened to possess lower mechanical properties as compared to the TPMS structure. This fact is supported by Al-Ketan et al. in their study since they stated that the TPMS structure exhibits excellent mechanical properties [72]. Nonetheless, Du Plessis et al. realized that there is not much significant difference when comparing the mechanical properties of TPMS structures and strut-based structures [90]. According to Guo et al., although there is not much difference in the mechanical properties of the TPMS and strut-based structures, the TPMS structure showed a more uniform and smooth transition of stress distribution [56]. From these studies, we can see that the TPMS structure kind of possess the same mechanical properties as the strut-based structures.

In the matter of bone ingrowth, the researchers would take permeability and specific surface area as a prediction tool [68]. The specific surface area helps in predicting the cell absorption area, while permeability indicated the ability of the scaffold to facilitate the transportation of oxygen, nutrients and waste. Schwarz D had the highest specific surface area, while Gyroid had the highest permeability [11,48,56,91]. Therefore, we can say that the Schwarz D and Gyroid might be the suitable TPMS for bone ingrowth. However, it needs to be furthered verified by biological experiments. Schwarz P has a high manufacturing accuracy as compared to other TPMS since it has the simplest geometry rather than the other.

In conclusion, we can say that the TPMS structure can be a suitable design for bone tissue scaffold. The stretching structure and bending structure both have different advantages. Stretching TPMS has excellent properties while the bending structure possesses high permeability properties. In general, these TPMS showed properties that are similar to that of the natural bone.

#### 2.3.2. Voronoi Tessellation

The Voronoi structure is said to be similar to the host tissue in terms of morphology. This is a need in bone tissue engineering since it should be able to copy the natural bone properties [92,93]. The Voronoi structure can be produced when a mesh structure is generated based on random discrete points, which are then connected and performed a network structure [94]. In 2010, Kou and Tan had introduced the Voronoi method by creating irregular and random scaffolds, which were merged with Voronoi cells [95]. They used B-spline curves in order to indicate the irregularly shaped pores’ boundaries. When this method was adopted, it can be seen that the shape of the scaffold was kind of similar to that of the shape of the bone structure [96]. After the Voronoi method has been proposed by Kou and Tan, researches related to the usage of the Voronoi method has been widened progressively, which includes the reverse engineering method that adopted computed tomography (CT) scan method to extract its data. Yang and Zhao stated that the Voronoi method could be used to recreate a bone-like-shaped scaffold by utilizing the data obtained from a computed tomography (CT) scan [67]. Although the Voronoi structure can be generated via the tessellation method, which employs some indices such as trabecular thickness and bone volume to total volume ratio, it is still time-consuming, long-cycle and mostly unrepeatable experiments [31,97,98].

In the computational method, the Finite Element Analysis was used to indicate the stress of the Voronoi structure. The study carried out by Wei et al. showed that the stress gradient of the Voronoi structure increase when the amount of porosity is low and vice versa [99]. In terms of fluid properties, Gomez et al. suggested that the Voronoi structure is depending on the amount of porosity and the bone surface area, which is very much favourable [31]. Therefore, it helps in bone ingrowth. Although the structure is having a good resemblance with the properties of the cancellous bone, Maliaris and Sarafis discovered that the intersection of struts was exposed to a stress change [100].

#### 2.3.3. Other Parametric Design

Besides the most common two designs of a parametric scaffold, there are also other parametric designs, which help in finding the suitable scaffold shape. This is due to the demand of the bone tissue engineering scaffold, which needs them to be able to possess excellent mechanical and fluidic properties in terms of permeability. Naturally, many structures in our environment possess high compressive strength. For example, Achrai and Wagner discovered that the turtle shell structure might help in producing a feasible scaffold design [101].

#### 2.3.4. Method of Anatomical Features (MAF)

In addition, the B-spline curve method had also been adopted by Vitkovic et al. when they produced a scaffold via reverse engineering method for mandible tissue scaffold. In their study, they identified the mathematical equation that is governing each point in the shape of the damaged bone. By doing this, the shape of the scaffold that resembled the damaged bone shape can easily be reproduced. They also found that at a certain amount of porosity, the mechanical properties of the scaffold can match with its host tissue [102].

Besides that, there is also a parametric method that can be defined as the new approach to describe the geometry of human bones, which is based on anatomical landmarks. Since the researchers found difficulties in tailoring the bone substitute with the geometry of host tissue for a specific patient, the Method of Anatomical Features (MAF) was introduced by Vidosav et al. in their paper [103,104]. For example, the anatomical landmarks for femur bone are the Centre of Femoral Head. MAF has been a huge aid in determining the 3D model of the bone by using reverse engineering. In addition, it is also reliable in producing the predictive model of the bone or simply known as a parametric model of the bone [105]. The Method of Anatomical Features (MAF) consists of a few steps that are necessary to obtain the parametric model, and the most important step is to define the Referential Geometrical Entities (RGEs). Referential Geometrical Entities (RGEs) can be defined as the basic prerequisite in order to develop a successful reverse engineering modelling of the human bone as well as the predictive model of the human bone [106]. Planes, axes, curves, surfaces and points are examples of RGEs. All of the elements of the human bone must be referred to as the defined RGEs. In a study conducted by Stojkovic et al., they carried out MAF on the femur bone of a human, and they defined some of the RGEs of the femur bone of a human [107]. Anterior–Posterior (A–P) plane and Lateral–Medial (L–M) plane are the crucial views that needed to be defined precisely in order to develop the reversed model of the human femur bone successfully.

**Figure 1:**
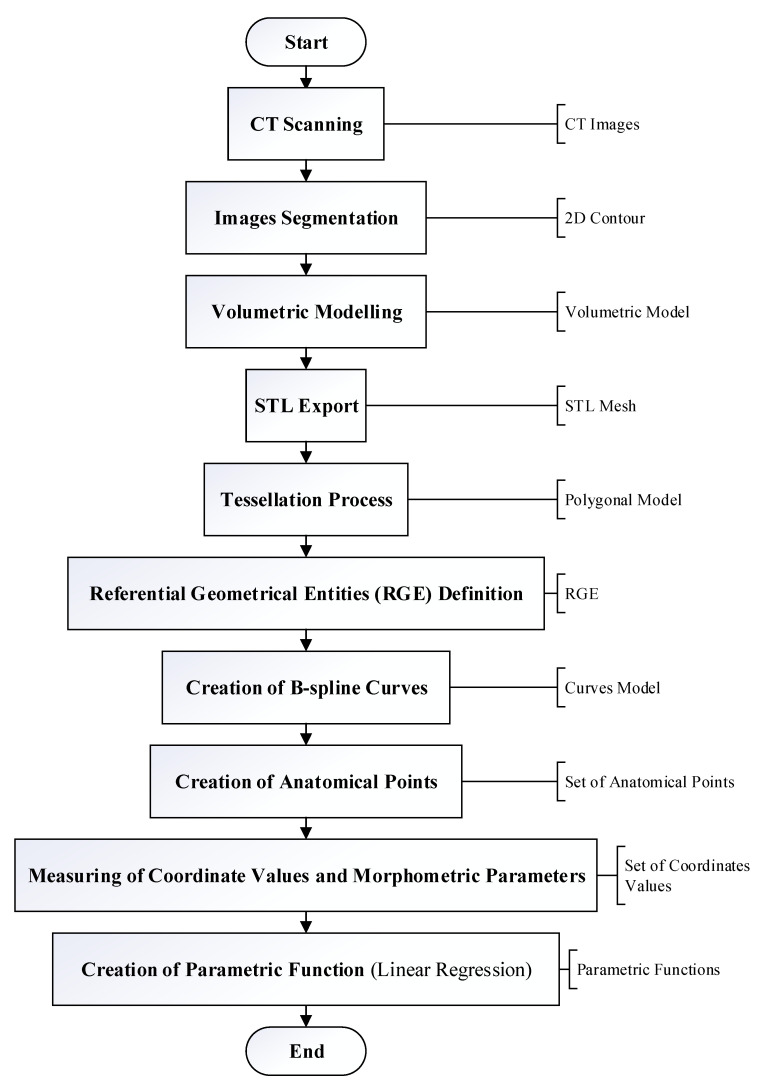
Flowchart of the Method of Anatomical Features (MAF).

In order to understand the procedure of generating a mathematical equation by using the Method of Anatomical Features (MAF), the following flowchart in Figure 1 can be referred.

**Step 1: CT Scanning**

In a real-world application, the CT scanning technique is used to identify any abnormalities of an organ. This method is carried out by scanning the organ so that the defective part of the organ can be detected.

**Step 2: Volumetric Modelling**

The volumetric model of the bone will be created in order to identify the initial geometry of the bone, which is required in order to locate the missing part of the bone. This model is created morphologically and anatomically in order to define the descriptive model of human bone. The model will then be saved in the STL file. The STL file will be exported into CAD software.

**Step 3: Tessellation Process**

This process is crucial in order to determine the polygonal model of the scanned bone. The tessellated model helps in identifying and filling any gaps that are found in the scanned bone during the STL mesh obtained.

**Step 4: Referential Geometrical Entities (RGE) Definition**

Referential Geometrical Entities or RGE includes the characteristics, points, planes, directions and views of the bone. These entities are defined in order to create a successful reverse engineering model of the bone.

**Step 5: Creation of B-Spline Curves**

These curves are created by using the referential geometrical entities (RGE) created earlier. However, a few additional curves might be needed in order to create curves that can fit the shape of anatomical features precisely.

**Step 6: Creation of Anatomical Points**

The anatomical points can be generated on the curves created in the previous step or can be created on anatomical landmarks. By creating the points on spline curves, they will be distributed evenly on the curves. Meanwhile, the points that are created on the anatomical landmarks will be positioned in correspondence to the landmarks such as the distal part of the femur. These points defined the boundary of the anatomical regions on the polygonal model. The process of defining RGE and the creation of B-spline and anatomical points are repeated for each part of the damaged bone.

**Step 7: Measuring of Coordinate Values and Morphometric Parameters**

Values of coordinates are measured on each part of the bone model in 3D. The morphometric parameters are also measured in the same 3D model.

**Step 8: Creation of Parametric Function (Linear Regression)**

The parametric functions can be generated by defining the relationship between morphometric parameters and coordinate values. The parametric model of the bone will be created, which consists of multiple parametric function. This model is used as the predictive model for the bone.

Generally, a parametric design is very much reliable in providing a feasible tissue engineering scaffold. Since a parametric design consists of a complex shape, it is very much compatible with the additive manufacturing sector. However, it is governed by a specific algorithm, therefore making it is difficult to be produced. Triply Periodic Minimal Surfaces (TPMS) is an excellent structure that provides the scaffold with an adequate amount of mechanical properties along while possessing a good amount of porosity. Meanwhile, a Voronoi structure is a top-notch structure that possesses excellent scaffold properties since it matches the properties of the host tissue and is able to imitate the actual structure of the host tissue.

### 2.4. Summary of Scaffold Design

Based on the previous discussion, there are a lot of unit cell scaffold designs that have been studied by the researchers. The designs can be categorized under various categories, which eventually bring many different benefits to each other. Table 4 summarizes the designs that have been adopted by the researchers in their studies.

Based on Table 4, the varieties of designs produced by the researchers has showed that the study that is involving the design of scaffold has been rapidly increasing especially with the aid of the emerging technologies. Non-parametric designs were chosen by the researchers previously due to their simpler design process as compared to the parametric designs. However, scaffold which possess a high amount of porosity will facilitate the tissue growth process, and it can be seen that the non-parametric designs can possess 60–80% porosity and exhibits lower elastic modulus values. Meanwhile, the parametric designs can increase their elastic modulus varies from 0.8 GPa up until 3.92 GPa with an adequate amount of porosity. This shows that the parametric designs can easily imitate the properties of the host tissue [108]. In addition, the usage of additive manufacturing and also 3D scanner has shown a great impact in contributing to the complex designs of scaffold structure. Therefore, there is a need to produce a design that is not just limited to imitating the bone structure, but also utilizing the designs that are available naturally.

## 3. Computational Software Used in Simulation of Tissue Engineering Scaffold

There is a lot of software that can be used to design and simulate the behaviour of the tissue engineering scaffold. The software can be utilized based on the function that is embedded in the software. Table 5 describes the characteristics of the software.

From Table 5, there are four common software programs that have been utilized by the researchers in order to determine the properties of the scaffold of bone tissue engineering. From the description, we can see that Solidworks and Catia belong to the modelling and designing part of the simulation process. The software programs are reliable in producing an accurate design of the scaffold, which then will be used in the properties’ simulation. Besides that, the Finite Element Analysis (FEA) is carried out mostly by using Abaqus software which is capable of visualizing the behaviour and failure mechanism of the scaffold model. Meanwhile, Ansys Fluent is used to simulate the Computational Fluid Dynamics (CFD) Analysis of the scaffold behaviour. It is able to simulate and visualize the scaffold behaviour under various conditions, especially in fluid flow analysis.

### Other Software

There is also other software that was used in simulating the behaviour of the scaffold mechanically and fluidic. For example, COMSOL Multiphysics was used by Uth et al. in their study in order to validate and optimize the design parameters of a scaffold [109]. Sahin et al. had also used COMSOL Multiphysics to carry out Finite Element Analysis (FEA) simulation [131]. Apart from that, Creo Simulate was also adopted by researchers since it is capable of designing a scaffold model [19,132]. It is also reliable performing a numerical analysis of an anatomical model [133].

From these trends, we can see that there is a variety of software that can be used to simulate the behaviour of the scaffold. However, the software chosen to be adopted in the study should match the objectives and able to carry out the desired simulation.

## 4. Challenges and Future Work Recommendation

With the 3D printing technology nowadays, it seems like computational methods have been attracting many researchers’ attention in producing many studies that can fully unleash the potential of computational modelling in the future. However, as we know, there are no technologies that are perfectly developed. In computational modelling, there are still challenges that needed to be solved by the researchers. The limitation of the usage of computational modelling in designing a feasible scaffold of tissue engineering includes.

- The accuracy of the simulation technique. A model that is designed through computational method tends to be simplified in the computer-aided design (CAD) software. The structure of the scaffolds might be not fully accurate when it comes to comparing the simulated model and fabricated parts.
- The simulation of the scaffold’s behaviour can only be done by simulating uniaxial loadings in most studies. However, in real-life conditions, the scaffolds can be subjected to many loadings that are much more complicated as compared to uniaxial loadings.
- The simulation can only focus on small-scale models [3]. This is due to the constraints that are involving the technologies, such as computer power and application simulating time.
- In the future, it is advisable if the research can contribute to:
- Increase the accuracy of the simulation when it comes to the fabrication process. This process can be achieved by adopting image-based modelling such as images from a 3D scanner.
- The need to simulate the scaffolds models under various types of loading is crucial since many loads can be exerted on it, physically.
- To expand the study on using simulation method that can reduce the effect of size of the small-scale scaffold model on the large-scale scaffold model.
- Improvise the 3D printing technique is crucial since it can affect the surface of the scaffold.
- The studies can integrate artificial intelligence in the computational method.

## 5. Conclusions

This paper has reviewed the studies that comprise the application of the computational method in the area of bone tissue engineering. The computational method can be used to simulate the properties of the scaffold of bone tissue engineering. Moreover, the simulation technique can also be used to predict the design of the scaffold model. In order to produce a scaffold with good mechanical properties, many studies have been carried out to simulate the mechanical properties of the scaffold. It is desired that the scaffold possesses high compressive strength so that it can withstand the load exerted on it when it is planted into the body of a human or an animal. Since the porosity and mechanical strength have an inversely proportional relationship, most researchers came out with integrating the optimization process and simulation process, which produced the optimal scaffold model with good mechanical and fluid properties. Furthermore, the design of the scaffold was also simulated by using computational software. The types of designs that can be generated by using the computational method have varied. From the discussion, we can see that the parametric designs have attracted researchers’ attention since it exhibits a good balance between mechanical and fluid properties of the scaffold. Moreover, the parametric designs had also shown huge potential in terms of imitating the properties of the host tissue. With this review, it can be concluded that the computational method has great potential to be adopted in future studies due to its ability to predict the properties of the scaffolds. Moreover, the computational method is less time-consuming and very much reliable than the conventional method.

## Figures and Tables

**Table 1:** Non-parametric design and its characteristics.

Non-Parametric Design	Description	Advantages	Disadvantages	Ref.
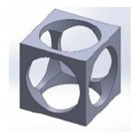 Circular [18]	A scaffold with a circular-shaped pore is a structure, which is commonly used in investigating the behaviour of the scaffold in terms of mechanical and fluidic.	Simple design—easy to be produced Less high-stress concentration points Exhibits stable resistance for fatigue damage Easy to fabricate using both conventional method and additive manufacturing	May cause underestimations of the behaviour of the scaffold High tendency to cause pore blockage, which affects bone growth by disrupting transportation of nutrients, oxygen and waste of the scaffold	[18,25,26,27,28,29,30,31,32,33]
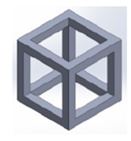 Square [18]	A square-shaped pore structure, which is reliable in producing high mechanical strength and adequate amount of porosity but also high in the stress concentration area	Simple design—easy to be produced Exhibits high proliferation rate Easy to fabricate using both conventional method and additive manufacturing	May cause underestimations of the behaviour of the scaffold Contains a high-stress concentration point	[19,29,34,35,36,37]
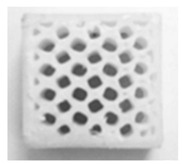 Honeycomb [38]	A structure that imitates the shape of the beeswax that exhibits excellent properties in terms of lightweight, stiffness and porosity	Simple design—easy to be produced Good mechanical stability Better at avoiding shrinkage of the scaffold during cell growth Promotes high cell proliferation	May cause underestimations of the behaviour of the scaffold Limitation on the fabrication based on the adjustable pore size, spatial arrangement and reproducible architectures	[21,39,40,41,42,43,44,45,46]

**Table 2:** Parametric design and its characteristics.

Parametric Design		Description	Advantages	Disadvantages	Ref.
Triply Periodic Minimal Surfaces (TPMS)	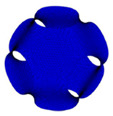 Schwarz P (Primitives)	Schwarz P (Primitives) is one of the earliest Triply Periodic Minimal Surfaces (TPMS) structures that was proposed by Schwarz in the 1860s. It belongs to the stretching surface structure. Its shape is strictly governed by a mathematical equation, as shown in Table 3.	Promotes high cell attachment, migration and proliferation Has the ability to possess the natural bone’s properties Helps in avoiding stress shielding High in mechanical strength as compared to the Schwarz D structure Possess a mechanical strength that complies to that of cortical bone when subjected to a low amount of porosity Simplest form of a TPMS structure	Complex shape that can only be generated through a mathematical equation Has a high concentration region in the neck of the structure Possess a low specific surface area as compared to the bending surfaces TPMS Can easily be fabricated via additive manufacturing, but not by using conventional method due to the complex shape	[51,52,53,54,55]
	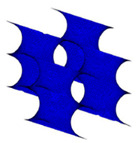 Schwarz D(Diamond)	Schwarz D (Diamond) can be categorised as a bending surface Triply Periodic Minimal Surfaces (TPMS) structure that was proposed by Schwarz in the 1860s. The Schwarz D shape can be generated via a mathematical equation, as shown in Table 3.	Promotes high cell attachment, migration and proliferation Has the ability to possess the natural bone’s propertiesHelps in avoiding stress shielding Possess a mechanical strength that complies to that of cortical bone when subjected to a low amount of porosity High in specific surface area as compared to the Schwarz P structure, thus it promotes high bone in growth rate Good structure for uniform stress distribution when subjected to ultimate pressure	Complex shape that can only be generated through a mathematical equation Low load-bearing capacity when subjected to uniaxial loading Can be easily fabricated via additive manufacturing, but not by using conventional method due to the complex shape	[51,52,55,56,57]
	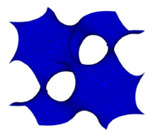 Gyroid	A gyroid structure was first introduced by Schoen, which became the most assessed Triply Periodic Minimal Surfaces (TPMS) structure in researches. Its shape is strictly governed by a mathematical equation, as shown in Table 3.	Promotes high cell attachment, migration and proliferation Has the ability to possess the natural bone’s properties Helps in avoiding stress shielding Mechanically better than a solid structure due to the consistency in the amount of porosity High in permeability, thus it promotes high bone in growth rate Good structure for uniform stress distribution when subjected to ultimate pressure	Complex shape that can only be generated through a mathematical equation Low load-bearing capacity when subjected to uniaxial loading Can easily be fabricated via additive manufacturing, but not by using conventional method due to the complex shape	[51,55,56,57,58]
	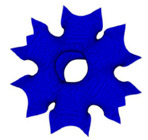 I-WP	An I-WP is one of the most assessed Triply Periodic Minimal Surfaces (TPMS) structure in research. The mathematical equation that is used to produce an I-WP structure can be found in Table 3	Promotes high cell attachment, migration and proliferation Has the ability to possess the natural bone’s properties Helps in avoiding stress shielding Possess excellent mechanical properties	Complex shape that can only be generated through a mathematical equation Has a high concentration region in the upper part of the structure Can be easily fabricated via additive manufacturing, but not by using conventional method due to the complex shape	[48,56,59,60,61]
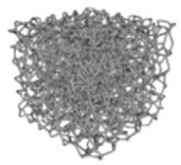 Voronoi [62]		A structure that is generated through a specific algorithm that creates random discrete points that turn into a network structure. The shape can easily imitate the structure of the host tissue. Therefore, it helps in bone ingrowth.	Has the ability to imitate the host tissue shape Matched the properties of the natural bone; thus, it helps in expediting the tissue growth rate Can be integrated with the usage of computed tomography (CT) scan; therefore, it has a good possibility to be used in real-life clinical application	Complex shape that is governed by a specific algorithm Time-consuming Might have difficulties in repeating the experiments Intersection of strut leads to stress changes	[63,64,65,66,67]

**Table 3:** Mathematical Equations of Triply Periodic Minimal Surfaces (TPMS) Structures.

TPMS Structure	Equation	Ref.
Schwarz P (Primitives)	cos(x) + cos(y) + cos(z) = t	[70,77]
Schwarz D (Diamond)	sin(x) sin(y)sin(z) + sin(x)cos(y)cos(z) + cos(x)sin(y)cos(z) + cos(x)cos(y)sin(z) = t	[77,78]
Neovius	3[cos(x) + cos(y) + cos(z)] + 4[cos(x)cos(y)cos(z)] = t	[70,77]
Gyroid	cos(x)sin(y) + cos(y)sin(z) + cos(z)sin(x) = t	[70,77,78]
I-WP	2[cos(x)cos(y) + cos(y)cos(z) + cos(z)cos(x)] − [cos(2x) + cos(2y) + cos(2z)] = t	[70,78]
Fisher-Koch S	cos(2x)sin(y)cos(z) + cos(x)cos(2y)sin(z) + sin(x)cos(y)cos(2z) = t	[76,78,79]
Fisher-Koch Y	cos(x)cos(y)cos(z) + sin(x)sin(y)sin(z) + sin(2x)sin(y) + sin(2y)sin(z) + sin(x)sin(2z) + sin(2x)cos(z) + cos(x)sin(2y) + cos(y)sin(2z) = t	[76,78]

**Table 4:** Scaffold design and its properties.

Type of Design			Material	Porosity (%)	Mechanical Properties			Software	Ref.
					Elastic Modulus (GPa)	Young’s Modulus (MPa)	Compressive Strength (MPa)		
Non-Parametric Design		Circular	Poly(L-lactic-co-glycolic acid) (PLGA), type I collagen, and nano-hydroxyapatite (nHA)	54.3–65.2	4.03–5.67	-	-	COMSOL Multiphysics	[109]
			Polylactic Acid (PLA)	80	-	-	0.163	Creo Simulate	[19]
			Poly-L-Lactic Acid (PLLA)	70–97	-	-	0.2–0.35	-	[110]
		Square	User-defined	64.8	-	0.5–1.0		Abaqus	[111]
			User-defined	60	0.16	-		Ansys Fluent	[36]
			Polylactic Acid (PLA)	80	-	-	0.186	Creo Simulate	[19]
			Polyamide (PA)	Graded 0.74–0.89	0.01	-	-	Abaqus	[54]
		Hexagonal	Poly-D-L-Lactic Acid (PDLLA)	55–70	-	274–1514	-	Ansys Fluent	[112]
			Glass Ceramic	60	2.4	-	-	-	[22]
					-	-	139	-	
		Octet	User-defined	60	6	-	-	Ansys	[36]
Parametric Design	Triply Periodic Minimal Surfaces (TPMS)	Schwarz P (Primitives)	Photopolymer Resin	30	-	150	-	Abaqus	[84,87]
			Photopolymer Resin	60	-	490	-	Abaqus	[84,87]
			Photopolymer Resin	Graded 30–60	-	350	-	Abaqus	[84,87]
			Visijet M3 Crystal	70	-	103.54	-	Abaqus	[113]
		Schwarz D(Diamond)	Photopolymer Resin	30	-	336	-	Abaqus	[84,87]
			Photopolymer Resin	60	-	79.5	-	Abaqus	[84,87]
			Visijet M3 Crystal	70	-	171.37	-	Abaqus	[113]
		Gyroid	Poly-D-L-Lactic Acid (PDLLA)	55–70	-	181–1011	-	Ansys Fluent	[112]
			Visijet M3 Crystal	70	-	145.05	-	Abaqus	[113]
		I-WP	Photopolymer Resin	Graded 40–60	-	170	-	Abaqus	[84,87]
	Voronoi		Poly-D-Lactic Acid (PDLA)	75–85	0.3–0.5	-	-	-	[31]
			Titanium Alloy	70	-	3920	-	Grasshopper	[114]
Other			Titanium Alloy	60–90	-	-	11.4 MPa	-	[115]
			Titanium Alloy	30–70	2.3–8.6	-	-	-	[116]

**Table 5:** Commonly Used Software for Tissue Engineering Scaffold Simulation.

Software	Description	Advantages	Disadvantages	Ref.
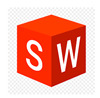 Solidworks	Computer-aided software that acts as a platform to design a scaffold model.	User-friendly interface Easy to be utilized Wider range of rendering options Capable of designing a parametric model Helps validate the products in terms of performance and safety	Lack of built-in library option Inefficient design in terms of model elements	[29,117,118,119,120]
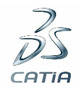 Catia	Computer-aided software that acts as a platform to design a scaffold model. It is also known to be a wide-ranging software that provides kinematic simulation.	Has a wider range of built-in library option Provides a kinematic solution Efficiently design elements of a model	Difficult to be immediately utilized by beginner Each program is dedicated to different industries	[73,102,121,122,123]
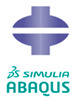 Abaqus	Abaqus helps in modelling and carries out a Finite Element Analysis. It facilitates in visualizing the behaviour of the scaffold design in terms of mechanical properties by providing the failure mechanism of the scaffold based on the boundary condition that has been subjected to the scaffold.	Able to simulate explicit/implicit model User-friendly interface Able to mesh a design accurately Excellent software for Finite Element Analysis (FEA)	Unable to modify orphan mesh Less reliable in simulating fluid properties as compared to Ansys Fluent	[35,113,124,125,126]
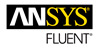 Ansys Fluent	Computer-aided engineering software that is reliable to simulate and visualize a Computational Fluid Dynamics Analysis (CFD). The scaffold can be subjected to various conditions that are including fluid flow analysis, etc. The software is capable of visualizing the behaviour of the scaffold in terms of fluidic properties.	Produce very good mesh properties User-friendly interface Excellent software for Computational Fluid Dynamics (CFD) Analysis	Limited mesh options Incapable of Finite Element Analysis (FEA) simulation without Computational Fluid Dynamics (CFD) Analysis	[127,128,129,130]

## Data Availability

No new data were created or analyzed in this study. Data sharing is not applicable to this article.
